# Testing the Mechanism of Action of Computerized Cognitive Training in Young Adults with Depression: Protocol for a Blinded, Randomized, Controlled Treatment Trial

**DOI:** 10.20900/jpbs.20200014

**Published:** 2020-06-19

**Authors:** Sara N. Rushia, Sophie Schiff, Dakota A. Egglefield, Jeffrey N. Motter, Alice Grinberg, Daniel G. Saldana, Al Amira Safa Shehab, Jin Fan, Joel R. Sneed

**Affiliations:** 1 Department of Psychology, The Graduate Center, City University of New York, New York, NY 10016, USA; 2 Department of Psychology, Queens College, City University of New York, Flushing, NY 11367, USA; 3 Department of Psychiatry, Columbia University Medical Center, New York, NY 10032, USA; 4 Division of Geriatric Psychiatry, New York State Psychiatric Institute, New York, NY 10032, USA

**Keywords:** computerized cognitive training, depression, magnetic resonance imaging, clinical trials

## Abstract

**Background:**

Depression is associated with a broad range of cognitive deficits, including processing speed (PS) and executive functioning (EF). Cognitive symptoms commonly persist with the resolution of affective symptoms and increase risk of relapse and recurrence. The cognitive control network is comprised of brain areas implicated in EF and mood regulatory functions. Prior research has demonstrated the effectiveness of computerized cognitive training (CCT) focused on PS and EF in mitigating both cognitive and affective symptoms of depression.

**Methods:**

Ninety participants aged 18–29 with a current diagnosis of major depressive disorder or persistent depressive disorder, or a Hamilton Depression Rating Scale score ≥12, will be randomized to either PS/EF CCT, verbal CCT, or waitlist control. Participants in the active groups will complete 15 min of training 5 days/week for 8 weeks. Clinical and neuropsychological assessments will be completed at baseline, week 4, week 8, and 3-month follow-up. Structural and functional magnetic resonance imaging (fMRI) will be completed at baseline and week 8. We will compare changes in mood, cognition, daily functioning, and fMRI data. We will explore cognitive control network functioning using resting-state and task-based fMRI.

**Results:**

Recruitment began in October 2019; we expect to finish recruitment by April 2022 and subsequently begin data analysis.

**Conclusions:**

This study is innovative in that it will include both active and waitlist control conditions and will explore changes in neural activation. Identifying the neural networks associated with improvements following CCT will allow for the development of more precise and effective interventions.

**Trial Registration:**

ClinicalTrials.gov NCT03869463; https://clinicaltrials.gov/ct2/show/NCT03869463.

## INTRODUCTION

Depression is a widespread public health issue with 20.2% of adults aged 18–29 reporting at least one episode of depression over the course of their lifetime, and 12.9% reporting at least one depressive episode in the past 12 months [1]. Depression is associated with affective symptoms as well as a range of impairments in social [2], occupational [3–5], and educational functioning [2], physical difficulties [6–8], and suicide [9]. Emerging adulthood (ages 18–29) represents a distinct transitional period in development with unique changes such as decreased family contact, the introduction of new significant friendships and romantic relationships, and increased financial responsibility [10]. Furthermore, the prevalence of depression and other psychiatric disorders is significantly higher in emerging adults than in any other age group [11]. Taken together, young adults aged 18–29 with depression represent an important, distinct population to target for novel treatment options.

The most common treatment for depression is antidepressant medication; however, only 60% of individuals achieve remission with their first treatment [12]. Additionally, depression falls on a continuum, and individuals with minor depressive symptoms who do not meet diagnostic criteria for major depressive disorder (MDD) still experience similar deficits in daily functioning [13–17]. Individuals who fall below the threshold of MDD diagnosis or who do not respond to antidepressants require novel interventions to address the associated impairments.

Depression is associated with a broad range of cognitive deficits in multiple domains including attention, verbal memory, processing speed, and executive functioning [18]. These cognitive deficits significantly affect the quality of life of those with depression and interfere with treatment of mood symptoms [19–22]. The existence and severity of the cognitive deficits are the strongest predictors of functional and treatment outcomes in depression [23–26]. The two most commonly and deleteriously impacted cognitive areas in depression are processing speed (PS) [19,27–29] and executive functioning (EF) [30–34]. Indeed, emerging adults with depression score significantly worse on tests of EF and PS than controls [32]. Processing speed is a lower-level, or fundamental cognitive resource, that facilitates the operations of higher-level (including executive) functions [35]. Existing treatments, such as antidepressants and psychotherapy, do not directly target these cognitive processes. This is important because targeting cognition may enable individuals to better benefit from interventions that target affective symptoms [19].

Computerized cognitive training (CCT) is an intervention that directly targets cognitive functions as a means to mitigate both the affective and cognitive symptoms associated with depression. CCT is comprised of exercises that strengthen specific cognitive processes via repeated practice [36]. Evidence of the effectiveness of CCT in improving cognitive functioning has been found in populations ranging from healthy adult populations [37,38] to diagnostic conditions including ADHD [39–41], schizophrenia [42], bipolar disorder [43], traumatic brain injury [44,45], minor neurocognitive disorder [46], and Alzheimer’s disease [47]. Beyond CCT’s effect on cognitive functioning, previous research has identified areas of improvement in other symptom domains following a course of CCT. Particularly, in a meta-analysis of CCT in depression, significant improvements were found in cognitive areas such as attention and working memory, as well as in affective symptomatology and everyday functioning, areas not directly targeted by CCT [48].

CCT specifically targeting processing speed (PS) may have particular benefits. In the Advanced Cognitive Training for Independent and Vital Elderly (ACTIVE) study focusing on older adults, PS-focused CCT was associated with significant reduction of 5-year mortality rates [49], risk of quality of life decline after 5 years [50], and greater likelihood of improvement in sense of personal control over one’s life after 5 years [51] when compared to other types of CCT. PS-focused training was also associated with reduced risk for depression after 1 year [49]. Similar findings have been reported in younger adults as well [52–57].

PS is of particular importance because decreased PS might prevent higher order mental operations from being executed properly. In depressed populations, previous research has found that PS mediates performance on tasks of executive function [28,58]. Given the relevance of PS in depressed populations, we propose a model of cognitive influence on mood regulation that places processing speed at the center, mediating the relationship between executive dysfunction and mood dysregulation [59]. With deficits in PS, information is processed too slowly, leading to the inability to perform executive functions. Failure to perform executive functions, such as inhibition, problem solving, planning, and flexibility, makes it difficult to regulate mood, which leads to increased rumination, worry, inability to suppress thoughts, and inability to select and modify adverse situations and behaviors.

In a preliminary study, we tested how different types of CCT may show different results [60]. When comparing PS/EF CCT to verbal-based CCT in young adults with depressive symptoms, the PS/EF group had greater gains in executive functioning and processing speed than the verbal group. Additionally, both groups saw significant and comparable improvements in self and clinician-rated depression severity and everyday functioning, though the PS/EF group obtained equivalent improvement in half the training time. Consistent with our model, PS/EF-targeted CCT may be more efficient than verbal ability-targeted CCT.

To elucidate the mechanism of action of CCT on mood, cognition, and everyday function, our proposed study will expand on the preliminary study by exploring the neural mechanism of action that underlies these improvements. Subserving EF and mood regulatory functions is the cognitive control network (CCN), an externally-oriented brain network comprised of two subnetworks, the frontoparietal network (FPN) and cingulo-opercular network (CON), and subcortical structures, including the thalamus and basal ganglia [61–63]. The FPN is composed of the frontal eye field, supplementary eye field, mid frontal gyrus, intraparietal sulcus, and superior parietal lobule [61,64]. The CON consists of the anterior cingulate cortex (ACC) and anterior insular cortex [65,66]. The CCN is ordinarily activated when engaged in a purposeful, goal-oriented task. Additionally, the CCN is involved in error detection and resolution [67], emotional processing [68], and information processing [69]. In young adults with depression, however, the CCN fails to come online when performing an executive task [70], and conversely, has greater functional connectivity at rest, indicating a failure of deactivation in the absence of cognitive effort [71]. Subthreshold depression has also been associated with impaired resting-state functional connectivity of the CCN [72]. This results in difficulties with attention, inhibitory control, novel generativity, planning, and working memory in individuals with depression [73,74]. Furthermore, fMRI studies using an emotionally-valenced go/no go task of behavioral inhibition and set shifting have shown different activation patterns in depressed patients as compared to healthy controls in areas such as the lateral orbitofrontal cortex, rostral ACC, and anterior medial prefrontal cortex [75], indicating an overlap with the CCN.

Given that non-specific cognitive training has been found to increase cortical thickness in the prefrontal cortex (PFC) [45], one distinct possibility is that CCT restores this neural network.

The CCN has purposes beyond cognitive processes alone. The CCN is connected structurally and functionally to other limbic areas (such as the amygdala, hippocampus, and dentate gyrus) acting as a regulator of affective states. Disruption in this cortico-limbic connection is thought to be central to mood dysregulation in young adults with depression [76]. Further, the magnitude of increased functional connectivity within the CCN when at rest significantly predicts the severity of depressive symptoms [70,77]. The CCN is also related to antidepressant treatment response. Poor antidepressant treatment response is associated with impaired functional connectivity of the CCN as well as damage to the ACC and dorsolateral prefrontal cortex (DLPFC) [78], whereas successful antidepressant treatment appears to alleviate deficits in the CCN [68]. Thus, the relationship between lack of response to antidepressant treatment and executive dysfunction is likely mediated by CCN integrity. PS/EF training may result in greater and more efficient improvement by directly targeting the CCN, with improvements in cognitive control and emotional processing corresponding to restorations of the dysfunctional network in depression. As such, there may be a greater increase in CCN activation and connectivity after CCT as compared to waitlist control, with a greater effect seen for PS/EF CCT than verbal CCT (active control).

Additionally, this study aims to address a major limitation of prior CCT research. While most CCT trials included a waitlist control condition, this study will include both active and waitlist control conditions. The active control condition will allow us to control for statistical regression to the mean, systematic selection biases, and non-specific effects of expectancy, engagement, motivation, and novelty associated with any type of engagement with cognitive training [79,80]. With the inclusion of the active control and waitlist control conditions, we will be able to isolate the effects of the PS/EF CCT and identify the neural mechanism of action that is specific to this type of training.

Thus, the primary aim of this study is to determine whether PS/EF CCT is superior to, and leads to faster improvement than, verbal ability CCT and waitlist control. The secondary aim of this study is to determine whether PS/EF CCT leads to greater change in CCN activation and connectivity than verbal ability CCT and waitlist control.

## METHODS

### Study Design Features and Rationale

Ninety young adults exhibiting mild to moderate depressive symptoms will be randomized in an 8-week computerized cognitive training (CCT) trial delivered via smartphone application. Mobile-based CCT was chosen as it is particularly relevant for young adults, with 92% of those aged 1829 owning a smartphone [81]. Mobile CCT is also consistent with the goal of personalizing medicine as it is easily accessible, inexpensive, noninvasive, and scaled to the skill level of each individual. Neuropsychological and clinical assessments will take place at Queens College, City University of New York, and fMRI will be conducted at The Graduate Center’s MRI facility, the Advanced Science Research Center. Participants will be randomized to one of three conditions: processing speed/executive functioning (PS/EF) CCT, verbal ability CCT (active control), or waitlist control (undergo assessment and imaging procedures and will be not be given access to the training program until 8-week completion). The randomization sequences will be balanced in blocks of random size to prevent researchers from guessing what the next participant’s condition assignment might be. In order to reduce expectancy bias, the informed consent signed by all participants prior to participation will not indicate which condition is the intervention, the active control, or the waitlist control. Instead, the consent will indicate that the participant will be assigned to one of three groups, two which target different aspects of brain functioning and the third which will begin cognitive training after 8 weeks.

### Role of Sponsor

The study is funded by a National Institute of Mental Health (NIMH) grant and internally supervised by a Data Safety Monitoring Plan. We customized a specific set of CCT training modules for participants in the PS/EF CCT condition and the verbal CCT condition using the mobile phone platform and application for cognitive brain training, Peak. These modules were also used in our preliminary study [60]. Over the course of this study, the Peak training modules will remain consistent for all participants. The current version of Peak is version 5.9; any updates that Peak makes to the application will not affect the specific games available to the research participants. Participants will access Peak on their mobile phones after they are provided with account information. Peak provides the research platform for free and does not have a role in the final study design, data interpretation, or publication of results. Participants will not be required to pay for the Peak account and will not have any post-study commitments to continuing to use Peak. None of the study team have any financial conflicts with Peak.

### Recruitment and Eligibility

Young adults with subthreshold depression, persistent depressive disorder, and mild to moderate major depressive disorder will be recruited from flyers posted in the community and local clinics, from self-referrals after participating in other studies for course credit at Queens College, and from an open-access website (i.e., Craigslist).

#### Inclusion/exclusion criteria

Detailed inclusion/exclusion criteria are described in Table 1. Notable inclusion criteria include a restricted, inclusive age range of 18–29; current diagnosis of major depressive disorder/persistent depressive disorder or an HDRS score ≥ 12; IQ score ≥ 85; daily access to a smartphone or tablet with internet connection; and willing and able to complete fMRI, mood, and neuropsychological testing. Intellectual functioning will be estimated using the Wechsler Test of Adult Reading (WTAR). The cutoff score of 85 was used to include individuals with IQ > 16th percentile in order to ensure that individuals have capacity to consent. This cutoff was also used in our preliminary study [60].

Participants will be excluded from participation if they lack English-speaking ability; have evidence of a major psychiatric, neurological, or medical disorder; severe depression (HDRS ≥ 30); active suicidal ideation, intent, plan, or have attempted suicide within the past year; use of medications that negatively impact cognition; history of drug or alcohol abuse or dependence within the past year; or regularly take part in online brain training (≥2 times per week). Participants will not be excluded for simultaneous engagement in psychotherapy or pharmacotherapy, or for use of medications that could be considered “cognitive enhancers” (e.g., Ritalin), as CCT may be considered a supplemental treatment that can be used in conjunction with evidence-based treatments for depression, until CCT has been established as a primary treatment. Furthermore, a recent meta-analysis of CCT in depression revealed that concurrent treatments did not affect the significant findings of small-moderate improvements in symptom severity and daily functioning and moderate-large effects on measures of EF [48]. However, once enrolled in the study, participants will be asked to refrain from beginning therapy or new medications, if possible, for the duration of the acute 8-week trial.

#### Depressive symptoms criteria

Depressive symptoms will be assessed at screening and all subsequent visits by the Hamilton Depression Rating Scale (HDRS). The central aim of this study is to demonstrate the mechanism of action of CCT, so in order to be as inclusive as possible of the depressive spectrum, inclusion criterion will be an HDRS score ≥ 12. Literature identifies mild forms of MDD as HDRS scores ranging from 8 to 16 [82]. Severe MDD (HDRS ≥ 30) will be excluded in order to ensure such individuals receive appropriate evidence-based treatment. The Beck Depression Inventory, Second Edition (BDI-II) will be used as a secondary measure of depressive symptoms. This self-report scale will be administered at all in-person visits and through an online survey format in weeks 2 and 6. The online administration of the BDI-II has similar psychometric properties as the in-person administration [83]. The HDRS and BDI-II were chosen over other depression rating scales to ensure compatibility of study results with other studies and to have both subject-rated and clinician-rated measures.

### Length of Clinical Trial

The length of this clinical trial will be 8 weeks. There is currently no consensus on the minimum amount of time spent in CCT required for therapeutic benefit, with training times ranging from 90 min [3,84] to over 1000 minutes [85] across successful programs for adults with depression. The choice of 8 weeks is also based on the success of an 8-week pilot study. In both the pilot study and the current study, the training protocol is 15 min per day × 5 days × 8 weeks, which equals 600 total minutes of training time. This amount appears to be more than sufficient in providing therapeutic benefit [60]. A follow-up visit will be conducted 3 months after the trial ends to determine lasting effects of CCT on mood, cognition, and daily functioning. Thus, the total study duration will be 20 weeks of participation. Since this study is considered low risk, harm from participating in this trial is not anticipated.

### Treatment Regimen

An initial pre-screening will be completed over the phone to assess initial eligibility criteria. Participants will provide information regarding age, access to smartphone/tablet, MRI contraindications, current/prior engagement in brain training games, and a brief medical history. If deemed eligible, participants will be invited for an in-person eligibility screen, which will assess for depressive symptoms and risk. Participants will be enrolled into the study after screening for eligibility and providing in-person informed consent. The randomization will be assigned by the statistician and then carried out by the unblinded research coordinator. Enrolled participants will engage in four clinic visits (weeks 0, 4, and 8, and 3-month follow-up) and receive two fMRIs (weeks 0 and 8). Participants will also complete an online version of the BDI-II during weeks 2 and 6. To protect study participants, if a participant shows a 20% increase from their baseline BDI-II score for two consecutive timepoints they will be discontinued from the study and provided with referrals for mental health services. This cut-off was made as a clinical decision based on data from our preliminary study [60] as well as research that shows that the minimal clinically important difference for improvement on the BDI-II is a 17.5% reduction in scores [86]. If a 17.5% reduction in BDI-II scores corresponds to a clinically important improvement, it is likely that a similar increase in scores would be a clinically important exacerbation. In our preliminary study [60], the mean baseline score on the BDI-II was 22.98 (SD = 11.58), so a 20% increase would be analogous to a shift from a score of 23 to a score of 28 for two consecutive timepoints. Additionally, BDI-II scores in general, as well as specific items, will be closely monitored. In particular, item 9 of the BDI-II, which assesses for suicidality, will be monitored for every BDI-II for each participant. If a participant at any time indicates an answer ≥ 1 on this item, we will have the participant complete a risk assessment using the Suicide Assessment Five-Step Evaluation and Triage (SAFE-T [87]) with Columbia Suicide Severity Rating Scale (C-SSRS [88]) Protocol, which describes the level of risk and subsequent response required by the research team.

#### Randomization

Before randomization, a blinded researcher will conduct the neuropsychological battery and the participant will complete the baseline fMRI. There will be one unblinded independent researcher who will conduct the randomization sequences in order to keep study researchers blinded to group. The randomization sequences are balanced in blocks of random size (2, 4) to prevent researchers from guessing what the next participant’s condition assignment might be. After the neuropsychological testing and fMRI have been completed, the unblinded researcher will assign a study ID to the participant, using a pre-populated form from the statistician’s randomization assignment, which will determine the study condition the participant will receive: PS/EF CCT, verbal CCT, or waitlist control. The Peak account information will be generated after the neuropsychological battery and fMRI have been completed. The unblinded researcher will then assist the participant in downloading the Peak application to their mobile phone or tablet. Participants will receive a specific Peak account and email using only their study IDs to ensure that their data is de-identified.

#### CCT modules

No training sessions are provided to the participants; participants are told to contact the research team if they have any questions regarding how to use the application or play the games, or if they have difficulty accessing the application. Peak does not provide technical support directly to the participants. Participants randomized to the PS/EF CCT group will play the following five modules: Must Sort, Face Switch, Refocus, Tunnel Trance, and True Color (descriptions of these games are found in Table 2). Participants will be able to choose which modules to play during each training session. The selected games will tap into processing speed and executive function skills by necessitating quick responses. Repeated practice of these skills over time that scales in difficulty in response to the participant’s performance drives improvement of these abilities. The selected games reflect the deficits characteristic of depression in young adults. The active control condition (verbal CCT) is identical to the PS/EF CCT condition in duration, training platform, and frequency of sessions. Participants in this condition will complete the following five modules that tap into verbal ability: Word-A-Like, Word Pairs, Worth Path, Word Fresh, and Babble Bots (descriptions of these games are found in Table 3). Participants will be able to choose which modules to play during each training session. Participants randomized to waitlist control will undergo all assessment and imaging procedures but will not receive CCT until after 8 weeks. Participants in the PS/EF CCT group and the verbal CCT group will spend the same amount of time on the platform during the training phase, which will consist of five 15-minute training sessions per week for 8 weeks (40 total training sessions). Participants will receive automatic feedback within the application about their scores and performance change over time after playing the games. Participants will have access to the games through cellular data or internet connection.

#### Clinic-based clinical, cognitive, and functional assessments

At the screening evaluation, trained research assistants will administer the depression section of the Anxiety Disorders Interview Schedule for DSM-IV (ADIS-IV), 21-item HDRS, and 21-item BDI-II to document depression severity and diagnosis. Participants will also complete the Sheehan Disability Scale (SDS) to determine daily functioning, and the WTAR used in conjunction with demographic variables to estimate IQ. At baseline (week 0), participants will complete a comprehensive clinical and neuropsychological assessment. Clinical and functional assessments administered in person include the HDRS, BDI-II, and SDS. The neuropsychological battery includes the following: Delis-Kaplan Executive Function System (DKEFS) Trail Making Test to assess PS and EF, specifically speeded attention and cognitive flexibility; DKEFS Color-Word Interference Test to assess PS and EF, specifically response inhibition and cognitive flexibility; Wechsler Adult Intelligence Scale, Fourth Edition (WAIS-IV) Digit Span Forwards, to assess immediate attention, and Digit Span Backwards, to assess working memory; WAIS-IV Coding and Symbol Search to assess PS; DKEFS Letter and Category Naming Test to assess verbal fluency; California Verbal Learning Test, Version 2 (CVLT-II) to assess verbal memory; and Brief Visuospatial Memory Test-Revised (BVMT-R) to assess visual memory. Additionally, participants will complete an expectancy scale at baseline which assesses how much they expect to improve during the intervention. Expectancy effects are important to assess given their potential impact on clinical trials research [89,90]. Participants will complete the User Engagement Scale to measure multiple aspects of engagement, usability, and satisfaction.

### Timeline of Longitudinal Assessments

At subsequent clinic visits (weeks 4 and 8 and 3-months), the same neuropsychological and clinical battery will be completed. Participants will complete the User Engagement Scale at week 8. The three-month follow-up visit will be to determine treatment gains and lasting effects of CCT on mood, cognition, and daily functioning.

### Study Measures

Study measures with time-points of administration are listed in Table 4 At screening, the ADIS-IV, HDRS, BDI-II, SDS, and WTAR will be administered, and current medications will be documented. The HDRS, BDI-II, and SDS will be administered again at weeks 0, 4, and 8, and 3-month follow-up. At weeks 0, 4, and 8, and 3-month follow-up, the neuropsychological battery will be administered, and current medications will be documented. At weeks 0 and 8, participants will undergo an MRI scan of the brain. The MRI scan will include the following sequences: T1 Magnetization-Prepared Rapid Gradient-Echo (MP-RAGE), as well as a task-based fMRI sequence consisting of the emotional go/no-go task, and pre-post task-based resting-state fMRI scans (see Table 5).

### Blinded Training Procedure

The blinded research assistant will administer the neuropsychological and clinical battery. The blinded research assistant will be blind to the group assignment of participants, but not to the aim of the research study. The unblinded research coordinator will be responsible for administering the initial Peak instructions and technical support. The unblinded research coordinator will be aware of group assignment and the aim of the research study. Twice per week, the unblinded research coordinator will review the compliance of all participants through the Peak application. The Peak application provides information about how many games each participant plays, which is then used to determine the amount of time the participant spent playing games. Noncompliant participants will be encouraged to participate unless otherwise notified. If there is a period of time where a participant does not train for three days in a row, the unblinded research assistant will call the participant to evaluate why training has not occurred and provide technical support if necessary, in order to ensure high compliance rates.

### Criteria for Early Discontinuation

Early discontinuation may occur due to one or more of the following reasons: (1) the participant’s decision not to continue the computerized training due to lack of time, interest, or motivation; (2) unavoidable circumstances (e.g., moving residence and unwilling or unable to return for in-person evaluations); (3) investigator decision to terminate; (4) death or prolonged hospitalization for medical reasons. We will not terminate participation for non-adherence. If non-adherence occurs, we will document the level of the participant’s adherence and still include the data in intent-to-treat analyses.

### Data Management

Data entry will be completed by graduate and undergraduate research assistants on the study protocol. Data entry will be done throughout the project. Data will be kept in a de-identified file kept on an encrypted password-protected external hard-drive that only research personnel have access to.

### Hypotheses

The primary aim of the study is to determine whether PS/EF-focused CCT is superior to, and leads to faster improvement than, verbal ability-focused CCT and waitlist control. Hypothesis 1: there will be greater improvement in mood, cognition, and everyday functioning in participants randomized to CCT (both PS/EF and verbal) compared to waitlist control. Hypothesis 2: there will be greater improvement in EF and PS outcomes in participants randomized to PS/EF CCT compared to verbal CCT and waitlist control. Hypothesis 3: there will be faster improvement in mood, cognition, and everyday functioning in participants randomized to PS/EF CCT compared to verbal CCT and waitlist control.

The secondary aim of the study is to determine whether PS/EF-focused CCT leads to greater change in CCN activation (task-based fMRI) and connectivity (resting state fMRI) than verbal ability-focused CCT and waitlist control. Hypothesis 1: there will be a greater increase in CCN activation and connectivity among participants randomized to PS/EF CCT and verbal CCT compared to waitlist control. Hypothesis 2: there will be a greater increase in CCN activation and connectivity among participants randomized to PS/EF CCT compared to verbal CCT. Hypothesis 3: clinical improvements will be positively associated with an increase in CCN activity and connectivity.

### Statistical Analysis and Sample Size

Prior to conducting any tests of hypotheses, all data will be reviewed for extreme values using boxplots, scatterplots, histograms, and normal probability plots. When distributional assumptions are not met, data will be transformed or nonparametric procedures will be used. Descriptive statistics will be used to characterize the sample with regard to demographic and clinical characteristics including but not limited to age, education, race/ethnicity, gender, and depression severity.

In this study, missing data patterns will be analyzed explicitly. Data will not be assumed to be missing completely at random (MCAR) or missing at random (MAR). Rather, this assumption will be explicitly tested predicting “missingness” (missing = 1 and not missing = 0) at all assessment points post-baseline with baseline predictors (e.g., clinical, demographic, neuropsychological, and MRI), which can be accomplished in a logistic regression framework. More advanced approaches to handling missing data such as multiple imputation [91] or maximum likelihood estimation will be used rather than older, more biased approaches such as mean substitution, listwise or casewise deletion, or linear regression [92].

#### Aim 1.

To test for differences in improvement between the three groups on the primary outcomes for mood (HDRS), everyday function (SDS), and executive function (TMT-4), we will use multilevel modeling [93–95]. Multilevel modeling is a powerful and flexible statistical procedure that estimates intercepts and slopes for each individual (level 1) as well as intercepts and slopes for the average individual (level 2) over time using Maximum Likelihood (ML) estimation [93–96]. All analyses will adjust for gender and race/ethnicity (centered at their respective means). Time will also be centered so that the intercept reflects scores at week 4 (middle of the trial). To test whether average scores at week 4 and rate of change differs among the groups (PS/EF CCT, verbal CCT, and waitlist control), dummy variable coding will be used with 1 indicating membership and 0 indicating non-membership. With each covariate centered at its mean, the intercept will correspond to the mean of the reference group at week 4 (waitlist control) and the slope will reflect the difference between the groups included in the model (PS/EF CCT, verbal CCT) and the reference group (dummy coded variable excluded from the model). Because time can be centered at any point in the trial, we will also be able to run models with time centered at weeks 0 and 8 so that the intercept reflects scores at these points in the trial. This will allow us to test the hypothesis that PS/EF-focused CCT is more efficient, and therefore leads to faster improvement, than verbal-focused CCT or waitlist control. The −2LL (−2 Log Likelihood) test will be used to test model fit [94,95].

#### Aim 2.

To determine whether PS/EF-focused CCT leads to greater CCN activation on the emotional go/no go task (primary fMRI outcome), we will conduct independent-sample t-test on the contrast images (i.e., correct no-go events minus correct go events for different emotional combinations and for the collapsed faced over face valence). The group differences in connectivity from the dynamic causal modeling (DCM) and from the resting-state CCN connectivity analyses will be examined. In addition, the correlation analysis between the pre and post-treatment brain activation and connectivity changes and the symptom improvement will be examined. To determine whether PS/EF-focused CCT leads to increased activation and connectivity in the CCN compared to verbal-focused CCT or waitlist control, we will use a regressed change model [96] on CCN connectivity index scores (secondary fMRI outcome). Analyzing two time-points using simple change scores can be problematic because subtracting the pre-score (Y1) from post-score (Y2) does not take unreliability into account. Reliability of change scores tends to be low and decreases as the correlation between the pre-test and post-test increase. Unreliability, therefore, tends to increase change scores from t1 to t2. Consequently, simple change scores tend to overcorrect the post-score (Y2) by the pre-score (Y1). This problem can be averted by adopting a regressed change procedure [96,97], which simply treats the pre-score (Y1) as a covariate effectively removing all correlation of the post-score (Y2) from the pre-score (Y1). In this particular instance, CCN connectivity index scores for each subject at t2 (week 8) will be included in the model as Y2 (outcome) and their CCN connectivity index scores at t1 (week 0) will be included in the model as the Y1 covariate. Treatment condition will also be dummy-coded in this model so that the intercept corresponds to the mean of the reference group (waitlist control) and the slope will reflect the difference between the groups included in the model (PS/EF CCT, verbal CCT) and the reference group, which is excluded from the model.

#### Power.

Our preliminary study, with a sample of 46 participants, found significant within-subjects improvement across all outcomes and significant between-group differences in measures of EF and PS, with medium to large effect sizes for all findings [60]. Thus, with a recruitment goal of 90 participants in the current study, we expect to have significantly more power than our previous study. The power analyses for the current study were conducted using the pwr [98] and longpower [99,100] packages in R [101]. To power the proposed study at the 0.8 level (3 group RCT) to detect a medium between-group effect size (α = 0.05, two-tailed) with 20% attrition (a reasonable estimate based on our previous study [60] and consistent with dropout rates in psychotherapy RCTs [102]) would require a total sample of 195 or 65 per group. While this is obviously not feasible in a 3-year study (2.5 years actual recruitment, including database preparation, cleaning, and publication time at the end of the study) supported by a small grant mechanism, our proposed sample is twice that of our prior study. Also, in a previous study looking at CCN activation using the emotional go/no go task, significant activation was found in a number of different areas of the CCN with only 24 participants [103]. With a proposed sample size of 90, we are confident about detecting medium to large effect sizes across our proposed cognitive and neural activation variables of interest.

## RESULTS

The NIMH grant for this protocol was received in April 2019 and study recruitment began in October 2019. We expect to finish recruitment by April 2022 and to begin data analysis once recruitment has been completed.

## ETHICS APPROVAL

This study was approved by the Queens College Institutional Review Board (IRB) in November 2018. All participants will be required to have the capacity to provide informed consent and sign the IRB-approved informed consent form. Local IRB and state regulations for consent will be followed. Participant confidentiality is maintained according to HIPAA law for potential and enrolled participants before, during, and after the trial. The cognitive testing results and the fMRI findings will not be released to the participants.

## CONCLUSION

This study is innovative in at least four distinct ways. Firstly, it investigates the mechanism of action of CCT using task-based and resting state fMRI. To our knowledge, no study has demonstrated activation of the brain in adults with depression using CCT. Targeting the CCN with novel interventions that aim to improve its functional connectivity has the potential to significantly contribute to the treatment of depression. Identifying the neural networks associated with improvements in mood following CCT will allow for the development of more precise and effective interventions. This is critical given that a large number of individuals do not or only partially benefit from antidepressant and/or psychological treatment. Secondly, it includes both active and waitlist control conditions, which will help to control for threats to validity. Thirdly, it utilizes a smartphone-based CCT intervention, which can be completed virtually anywhere creating fewer disruptions in the participants’ life. Compared to existing treatments, it is easily accessible, inexpensive, noninvasive, and scales in skill level for each individual. Our study, therefore, is consistent with the goal of personalized medicine. Finally, this study includes an assessment of everyday function thereby assessing both near and far transfer. This is crucial as most CCT studies do not evaluate whether improvements on cognitive measures occur alongside or result in improvements in everyday functioning and quality of life [104–108]. Such assessments are necessary to evaluate claims that CCT only “teaches to the test” (i.e., CCT games are similar to outcome measures) and does not improve daily functioning.

The results will also help inform the design of a more powerful randomized controlled trial in many ways: determine sample size for a larger trial, identify domains and exercises most likely to improve, learn how subjects engage, identify gender effects, evaluate long-term benefits, and understand brain networks affected.

## Figures and Tables

**Table 1.** Inclusion/Exclusion Criteria.

**Inclusion Criteria**
1. Males and females ages 18–29 (inclusive) at the time of informed consent.
2. Current diagnosis of MDD/persistent depressive disorder or HDRS ≥ 12.
3. Daily access to smartphone or tablet with internet connection for the study duration.
4. Willing and able to complete fMRI, mood, and neuropsychological testing.
5. IQ ≥ 85.

**Exclusion Criteria**
1. Lacks English-speaking ability as determined by self-report and clinical evaluation.
2. Evidence of schizophrenia, schizoaffective disorder, psychosis, bipolar I or II disorder.
3. Active suicidal ideation, intent, or plan, or past suicide attempt within 1 year.
4. Severe depression (HDRS ≥ 30).
5. Neurological disorder (epilepsy, multiple sclerosis, traumatic brain injury, clinical stroke).
6. Use of medications known to have negative impact on cognition (benzodiazepines and lorazepam equivalents > 1 mg daily, narcotics, or anticholinergics).
7. History of alcohol or drug abuse or dependence within the past year.
8. Acute, severe, unstable medical illness. For cancer, acutely ill patients (including those with metastases) are excluded, but past history of successfully treated cancer does not result in exclusion.
9. Regular online brain training or regular player of non-fluency verbal games, defined as doing these procedures at a frequency of twice weekly or greater during the year prior to screening.

**Table 2.** PS/EF CCT games *.

Game Name	Description
Must Sort	Sort the items correctly by tapping on the left or right side of the screen. Wrongly scored items add a time penalty.
Face Switch	Determine if the woman on the top card is happy or if the man on the bottom card is wearing glasses.
Refocus	Determine if the number on the top card is even or if the letter on the bottom card is a vowel. Memorize the questions before they disappear.
Tunnel Trance	Compare the shape on the screen with the one displayed two shapes back. Memorize the second shape and determine if the current shape matches the shape presented two steps before.
True Color	Determine if the word at the top of a card matches the color of the word on the bottom.

* adapted from [60] with permission, copyright © Elsevier B.V.

**Table 3.** Verbal CCT games *.

Game Name	Description
Word-A-Like	Find words that are associated to the target. Tap the trashcan button to remove all letters from selected word.
Word Pairs	Pair words according to the rule by dragging and dropping clouds to combine.
Word Path	Create words to connect the yellow end points. Words must be spelled left to right or top to bottom and must use letters already placed or cover a yellow end point.
Word Fresh	Create words by swiping through adjacent letters. Words can be created in any direction.
Babble Bots	Create words of three letters or more by tapping the letters and pressing “submit”.

* adapted from [60] with permission, copyright © Elsevier B.V.

**Table 4.** Timeline of longitudinal assessments.

Measure	Screen	Baseline/Week 0	Week 4	Week 8	3-month follow-up
**ADIS-IV**	×				
**HDRS**	×	×	×	×	×
**BDI-II**	×	×	×	×	×
**SDS**	×	×	×	×	×
**WTAR**	×				
**Trail Making Test**		×	×	×	×
**Digit Span**		×	×	×	×
**Coding**		×	×	×	×
**Symbol Search**		×	×	×	×
**Letter Naming**		×	×	×	×
**Category Naming**		×	×	×	×
**Color-Word**		×	×	×	×
**CVLT-II**		×	×	×	×
**BVMT-R**		×	×	×	×
**Medication Check**	×	×	×	×	×

**Table 5.** MRI and fMRI sequencing information.

Sequence	TR/TE/TI	Flip Angle	Resolution	FOV	Acquisition Time
**T1-MPRAGE**	2000/4.68/965	9	1 mm iso	256 × 312 × 192 mm	6′48″
**Resting state fMRI**	1500/30	73	2 mm iso	256 × 256 × 132 mm	4′
**Task-based fMRI**	1500/27	73	2 mm iso	256 × 256 × 132 mm	25′12″
**Resting state fMRI**	1500/30	73	2 mm iso	256 × 256 × 132 mm	4′
